# Editorial for Special Issue “Brain Tumor Microenvironment”

**DOI:** 10.3390/cancers16223864

**Published:** 2024-11-18

**Authors:** Gianluca Trevisi, Annunziato Mangiola

**Affiliations:** 1 Department of Neurosciences, Imaging and Clinical Sciences, G. D’Annunzio University Chieti-Pescara, 66100 Chieti, Italy; 2 Neurosurgical Unit, Ospedale Spirito Santo, 65122 Pescara, Italy

The tumor microenvironment (TME) is a complex interplay of cells, extracellular matrix, and signaling molecules that significantly influences tumor growth, invasion, and resistance to therapy. This Special Issue delves into the intricate relationship between brain tumors and their surrounding microenvironment, shedding light on critical factors that drive tumor progression and resistance to treatment.

A comprehensive understanding of the TME is crucial for developing effective therapeutic strategies. Xiao et al. [1] highlight the heterogeneity of the TME in brainstem gliomas, a particularly challenging type of brain tumor. By classifying these tumors based on their TME characteristics, this study provides valuable insights for developing targeted therapies that can exploit the specific vulnerabilities of each TME subtype.

Glioblastoma, the most aggressive primary brain tumor, is one of the main focuses of this issue. Nickl et al. [2] explore the use of patient-derived tumor models to study the TME. These models offer a unique opportunity to understand the complex interplay between tumor cells and their microenvironment, including the role of immune cells, stromal cells, and extracellular matrix components. By studying these interactions, researchers can identify potential therapeutic targets and develop novel strategies to overcome treatment resistance.

Extracellular vesicles (EVs) are emerging as key players in cancer progression [3]. Jackson et al. [4] demonstrate how EVs secreted by medulloblastoma cells can promote tumor metastasis by activating specific proteins on their surface, such as matrix metalloproteinases. Targeting these EVs may offer a promising approach to prevent tumor spread and improve patient outcomes.

Artificial intelligence is revolutionizing cancer research. Alturki et al. [5] present a novel method for classifying brain tumors using deep learning techniques. By analyzing large datasets of imaging and genomic data, this approach can identify subtle patterns that may be missed by traditional methods, leading to a more accurate and earlier diagnosis of brain tumors.

Panigrahy et al. [6] explore the use of magnetic resonance spectroscopy (MRS) to monitor treatment response in pediatric diffuse intrinsic pontine gliomas (DIPG). By measuring metabolic changes within the tumor, MRS may provide valuable insights into tumor progression and treatment effectiveness. This non-invasive technique can help clinicians assess treatment response and adjust treatment plans accordingly.

Brosch et al. [7] investigate the role of sugar transporters in glioblastoma cell migration. By understanding how these transporters fuel tumor cell movement, we may be able to develop novel strategies to inhibit tumor invasion and metastasis. Targeting these transporters may offer a promising approach to limit tumor spread and improve patient survival.

Genoud et al. [8] provide a comprehensive review of current therapeutic strategies for glioblastoma, emphasizing the importance of targeting the TME. By understanding the complex interactions between tumor cells and their microenvironment, we can develop more effective treatments. This review highlights the potential of targeting immune cells, stromal cells, and signaling pathways within the TME to improve treatment outcomes.

The peritumoral brain zone (PBZ) is a critical region surrounding the tumor core that plays a significant role in tumor recurrence. Trevisi and Mangiola [9] review the biology of the PBZ, highlighting its unique microenvironment and its impact on tumor progression. Targeting the PBZ may offer a promising strategy to prevent tumor recurrence and improve patient survival.

Hypoxia, a condition characterized by low oxygen levels, is a common feature of solid tumors, including brain tumors. Shi et al. [10] review the role of hypoxia and cancer stem cells in glioblastoma development. Hypoxia can promote the survival and self-renewal of cancer stem cells, which are thought to be resistant to treatment and contribute to tumor recurrence. Targeting hypoxia and cancer stem cells may offer a novel approach to improve treatment outcomes.

The extracellular matrix (ECM) provides structural support and regulates cell behavior. Marino et al. [11] review the role of the ECM in glioblastoma, highlighting its modifications in the tumor microenvironment and its impact on tumor progression. Targeting the ECM may offer a promising strategy to inhibit tumor invasion and metastasis.

In conclusion, this Special Issue highlights the critical role of the TME in brain tumor progression. By understanding the complex interactions between tumor cells and their microenvironment, we can develop more effective therapies to improve patient outcomes. Future research should continue to investigate the TME and identify novel therapeutic targets to combat brain tumors.
